# The Design, Development, and Implementation of a Web-Enabled Informatics Platform to Enhance the Well-being of Individuals Aged 18-24 Years: Protocol for an Experimental Study

**DOI:** 10.2196/38632

**Published:** 2023-03-03

**Authors:** Bhavya Malhotra, Jagannath Sahoo, Mansi Gupta, Ashish Joshi

**Affiliations:** 1 Dehradun Institute of Technology University Dehradun India; 2 Foundation of Healthcare Technologies Society New Delhi India; 3 Graduate School of Public Health and Health Policy, City University of New York New York, NY United States

**Keywords:** well-being, mental health, nutrition, youth, digital health, interventions, India, health, lifestyle

## Abstract

**Background:**

Well-being is multidimensional, complex, and dynamic in nature. It is an amalgam of physical and mental health, essential for disease prevention and the promotion of a healthy life.

**Objective:**

This study aims to explore the features that impact the well-being of individuals between 18 and 24 years of age in an Indian setting. It further aims to design, develop, and evaluate the usefulness and effectiveness of a web-based informatics platform or stand-alone intervention to enhance the well-being of individuals aged 18-24 years in an Indian setting.

**Methods:**

This study follows a mixed method approach to identify factors influencing the well-being of individuals in the age group of 18-24 years in an Indian setting. The college-going students in this age group from the states of Uttarakhand (urban settings of Dehradun) and Uttar Pradesh (urban settings of Meerut) will be enrolled. They will be randomly allocated to the control and intervention groups. The participants in the intervention group will have access to the web-based well-being platform.

**Results:**

This study will examine the factors that influence the well-being of individuals aged 18-24 years. It will also facilitate the design and development of the web-based platform or stand-alone intervention, which will enhance the well-being of individuals in the age group of 18-24 years in an Indian setting. Furthermore, the results of this study will help generate a well-being index for individuals to plan tailored interventions. The 60 in-depth interviews have been conducted as of September 30, 2022.

**Conclusions:**

The study will help understand the factors that influence the well-being of individuals. The findings of this study will help in the design and development of the web-based platform or stand-alone intervention to enhance the well-being of individuals in the age group of 18-24 years in an Indian setting.

**International Registered Report Identifier (IRRID):**

PRR1-10.2196/38632

## Introduction

### Background and Rationale

Well-being combines physical and mental health, essential for disease prevention and the promotion of a healthy life. It is complex and multidimensional in nature. Well-being consists of objective and subjective measures. The objective measures indicate the quality of life, whereas the subjective measures comprise spiritual, social, psychological, affective, and cognitive judgments [1].

Psychological well-being refers to the psychological aspect. It consists of hedonic well-being (eg, the feeling of happiness), evaluative well-being (eg, contentment from life), eudaimonic well-being (eg, self-actualization, to know the meaning of life), and the other aspects of feeling well (eg, optimism) [1].

Mental health is described as a condition of well-being in which the person understands his or her ability to manage the usual pressures of life, can work effectively and productively, and can also contribute to the community [2]. As per Global Health Estimates 2016, the leading cause of nonfatal disease is mental and substance use disorders [3]. In the United States in 2017, more than 46.6 million adults were reported to have mental illness in the previous year [4]. In the entire world, in 2017, mental health was the second leading cause of the burden of disease in terms of years lived with disability and the sixth main factor of disability-adjusted life-years, which is a substantial threat to health systems, especially in low- and middle-income countries. Mental health is considered a crucial factor around the world; therefore, it is included in health policies and Sustainable Development Goals [5].

One-fourth of the entire world population is between 10 and 24 years of age, and in South Asia and Africa, 1 in 3 people are young. Mental health problems range from 10% to 20% worldwide among children and young people [6]. A study in 27 countries assessed the pooled prevalence of global mental health problems to be 13.4% among children and young people [7]. Disruptive behavior was commonly observed among children and young people. It is estimated that, in young people aged 16-24 years, 1 in 4 has experienced a minimum of 1 mental health issue in the previous year. The literature has shown that young people worldwide are less aware of mental health. To promote the mental well-being of young individuals, there is a need to educate them, raise awareness, and develop interventions [8].

As per the Registrar General of India, 34.8% of India’s population is between 15 and 34 years of age, and it is projected to be 34.1% by 2021. In India, in 2017, the prevalence of mental disorders was 197.3 million, which comprised 45.7 million people with depressive disorders and 44.9 million with anxiety disorders [9]. This was due to an increase in issues like nutritional disorders, phubbing, sedentary lifestyles, social media addiction, and substance abuse in lower-income countries. A study has been carried out in rural India to find the effectiveness of delivering evidence-based psychological treatment for depression by training community health workers [10]. One of the prior studies has shown an association between unhealthy eating and anxiety, stress, and depression in university students [11].

Research has demonstrated the usefulness and effectiveness of web-based interventions in youth with mental health issues [8]. It has been seen that young people feel more comfortable gathering mental health information through the internet for sensitive and personal matters. Therefore, studies recommend that there be a need to promote personalized digital health interventions (DHIs). The intervention should have the ability to engage individuals by sending personalized messages, reminders, or alerts to increase motivation. Research has also shown that technology acceptance and user engagement can be increased by using pictures and interactive content [12].

The findings of one study depicted that, to promote well-being to a significant population level, there is a need for intervention and educational programs in all segments of society [13].

However, there is limited literature on DHIs to improve the well-being of youth in India [14,15].

### Study Objectives

The following are the proposed study objectives:

1. To examine factors that influence the well-being of individuals aged 18-24 years in an Indian setting
2. To design and develop the web-based platform or stand-alone intervention to enhance the well-being of individuals in the age group of 18-24 years in an Indian setting
3. To evaluate the usefulness and effectiveness of the proposed informatics-enabled decision aid platform to enhance the well-being of individuals aged 18-24 years in an Indian setting

## Methods

### Study Design and Population

A mixed methods approach will be adopted that uses both qualitative and quantitative data to identify factors influencing the well-being of individuals in the age group of 18-24 years in an Indian setting. In this study, 100 individuals will be enrolled each in the intervention and control group at Uttarakhand and Uttar Pradesh colleges.

We will design and develop the proposed platform using human-centered design principles. Individuals will be randomly allocated to an intervention group (access to the proposed well-being platform) and a control group (standard education). The standard educational material will be mailed to the participants in pdf format on the following topics:

- Importance of physical activity
- Role of a balanced diet
- How to cope with depression, anxiety, and stress
- Steps to lead a healthy life

### Study Setting

We will recruit college-going students aged 18-24 years from the states of Uttarakhand (urban Dehradun) and Uttar Pradesh (urban Meerut).

### Study Eligibility

The inclusion criteria are as follows: (1) individuals 18-24 years of age, (2) of any sex (male or female), and (3) willing to give informed written consent.

**Figure 1:**
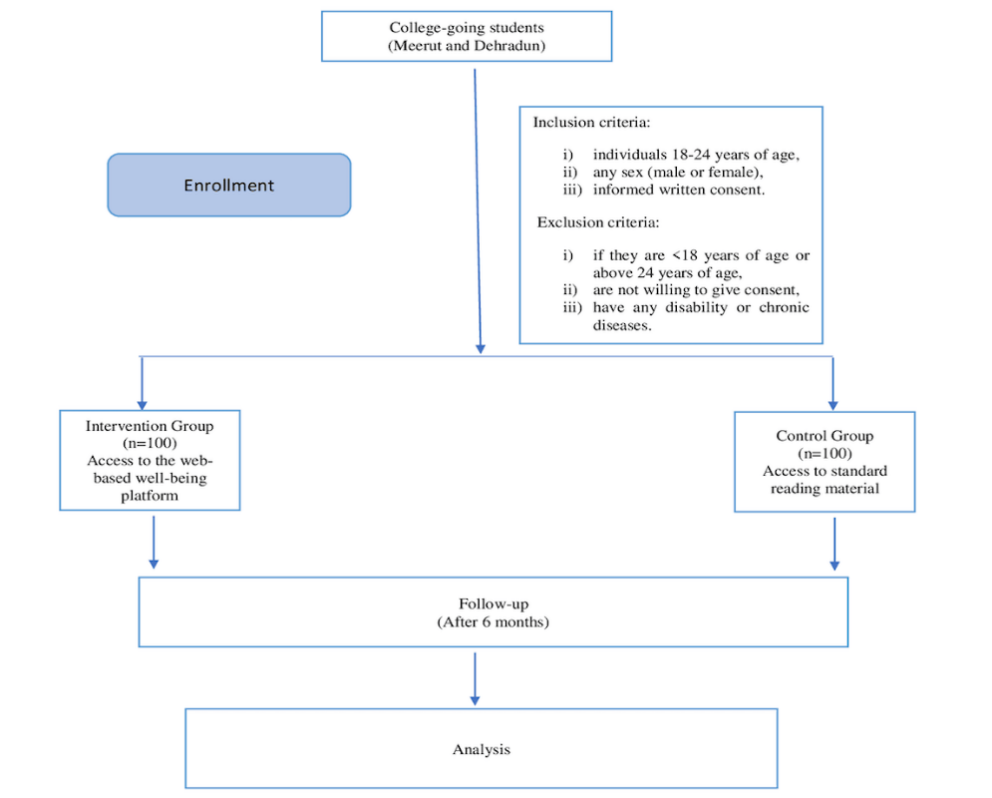
Schematic illustration of the methodology.

Participants will be excluded if they are younger than 18 years or older than 24 years, are not willing to give consent, or have any disability or chronic diseases. Figure 1 shows a schematic illustration of the methodology.

This study will follow a mixed methods pre- and postapproach for data collection using electronic data collection tools for qualitative and quantitative data from the study participants.

### Achieving Objective 1

We will conduct 60 in-depth interviews by enrolling participants from Dehradun and Meerut. These interviews will be conducted through Zoom meetings, and the sessions will be recorded after having consent from the participants. Both quantitative and qualitative data will be gathered from the participants. In the interview, the participants will be asked questions to assess their awareness of well-being, the challenges they come across, how they deal with them, and the impact of COVID-19 on their well-being. Each interview will take around 15-45 minutes.

The data collected will help us examine the factors that influence the well-being of students in the age group of 18-24 years. Emphasis will also be placed on understanding individuals’ prior knowledge and attitude related to stress, unhealthy diet, and physical inactivity.

The data will be gathered on the following variables: sociodemographic profile, medical history, and anthropometric status; familiarity with technology; health behavior; Depression, Anxiety, and Stress Scale (DASS-21); General Self-Efficacy Scale; psychological well-being scales; global physical activity; and dietary diversity.

In addition, content analysis of the qualitative data will help generate critical themes on the well-being of students in the age group of 18-24 years in an Indian setting.

### Achieving Objective 2

We will use principles of a human-centered approach to design and develop a web-based informatics platform to improve the well-being of individuals in the age group of 18-24 years in an Indian setting.

The usability of the web-based informatics platform will be evaluated by individuals who will use it and will provide feedback. The literature has shown that a 10-item Likert scale questionnaire called System Usability Scale calculates the usability of websites, mobile devices, hardware, software, and other technological applications. Based on the feedback on usability evaluation, system modification can be done. Finally, the proposed web-based well-being platform will be implemented in a randomized controlled trial to evaluate its usefulness and effectiveness.

### Achieving Objective 3

We will perform a randomized controlled trial with 100 college-going students from Dehradun and Meerut each in the intervention and control group. So the total number of students enrolled will be 200. The students will be randomly allocated to either a control group or an intervention group. The students in the intervention group will have access to the web-based well-being platform. A study was carried out to evaluate the efficacy of a web- and mobile-based intervention on college-going students (n=150) [16].

The data will be collected on the following variables: sociodemographics, health behavior, health literacy, depression, stress, anxiety assessments, general efficacy, physical activity, and dietary pattern at baseline and a follow-up at 6 months.

### Study Groups

The study participants will be randomly allocated either to the intervention group or the control group.

#### Intervention Group

The participants in the intervention group will have access to the web-based well-being platform. It can be accessed from an Android smartphone or web-based platform. The proposed platform will have the following components: (1) screening component, (2) learning component, and (3) evaluation component.

- Screening component: Variables recorded in the screening component include sociodemographics; health behavior (eg, physical activity levels and anthropometric assessments); health literacy; family history; familiarity with the use of technology; well-being scale; depression, stress, and anxiety assessments; and self-efficacy levels. In the screening component of the intervention, we will evaluate the health literacy level according to the education that will be given to the individual. Based on the data collected, a well-being index will be constructed to reflect on the variables attributing to the well-being of the youth. As the literature shows, well-being is dependent on various factors and is affected by the physical, social, and economic environment, life experience in every stage, family, poverty, and activity pattern [17,18].
- Learning component: Based on the well-being index generated for each individual, tailored messaging will be done using a series of culturally and contextually relevant messages. In this component, the individuals will get personalized feedback after interpreting the information gathered on all the parameters asked. In addition, they will receive weekly reminder messages to follow healthy behavior for their excellent well-being.
- Evaluation component: We will assess the usefulness and acceptance of the system using the system usability scale and monitor the usage. In addition, we will determine youth well-being using the well-being scale at baseline and a follow-up at 6 months.

### Informed Consent

The ethics committee of the university has approved the informed consent form. The research team will obtain the consent from all the individuals participating in the study. The research team will also explain them the analysis, duration, and need of the study. Individuals who are willing to participate will be asked to sign the consent form, and a copy of the consent form with study details will be given to them. The study is on college-going students, so everyone will be able to read and write. The research team is aware of both English and Hindi language, so they will be able to understand the response. The data captured will be stored safely and protected. The confidentiality of data will be maintained. The participants will be allowed to withdraw from the ongoing study anytime by giving reasons to withdraw. All the data gathered, including those from the participants who will withdraw, will be reported for the final analysis. No monetary compensation will be given to the participants enrolled in the study. The voluntary participation and time of the participants will be respected.

### Data Collection, Data Entry, and Quality Assurance

The data will be collected and entered by following standard techniques and protocols. Complete confidentiality of the information obtained from the study participants will be ensured. Frequent backups will retain all the data gathered, and all computers and specific data files will be password protected and kept in a locked file cabinet.

### Variable Assessment

#### Sociodemographic and Family History

The questionnaire included questions regarding the demographic factors of the respondents, like age, sex, family history, educational status, occupation, and income levels.

#### Health Behavior

Four crucial behavioral risk factors will be collected: tobacco consumption, alcohol consumption, impact of COVID-19, and sleep duration.

#### Familiarity With Technology

Individual assessment will include access to the type of smartphone, familiarity with the use of the internet, and knowledge of texting.

#### Anthropometric Measurements

Variables assessed include duration of diabetes, recent blood sugar report, type of medication, and height and weight measures that would help calculate BMI.

The measures in Textbox 1 will be administered.

**Measures to be used.**

**Sociodemographic and family history**

Age; sex; educational status; religion; income, if any; parents’ occupation

**Health literacy**

Rapid Estimate of Adult Literacy in Medicine scale

**Familiarity with technology**

Access to the smartphone and internet and knowledge of texting

**Health behavior**

Information on smoking, alcohol, physical activity, and substance abuse disorders

**Well-being scale**

Ryff scale

**Depression, stress, and anxiety assessments**

Depression, Anxiety, and Stress Scale

**Self-efficacy**

General Self-Efficacy Scale

**Dietary diversity**

Food and Nutrition Technical Assistance Diet Diversity

**Anthropometric measurements**

Height, weight, BMI

**Physical activity**

Global Physical Activity Questionnaire

**System usability**

System Usability Scale

### Data Security and Privacy

The data security will be done through regular backups, and all computers and specific data files will be password protected and kept in a locked file cabinet. In addition, the computers in which data is stored will be password protected and safely stored for 3 years after the completion of the study.

### Outcomes

The well-being index generated from this study can be used in other geographic regions for a similar population. If the youth of a nation is healthy, it will lead to the country’s growth.

The primary outcome of the study will be development of the student well-being index among urban settings. This will also facilitate the understanding of the various factors that affect the student well-being.

The secondary outcome of this study will further help us to understand the relationship between sociodemographic factors with dietary diversity, physical activity, depression, stress, and anxiety among college-going students.

A cross-sectional study was conducted, and the results showed significant associations between physical activity levels and sleep quality with mental health. The mental health issues are the same in both male and female college students [19].

The outcomes will be assessed by using the following tools.

#### Health Literacy

Health literacy is defined as the cognitive and social skills that determine the motivation and ability of individuals to gain access to, understand, and use information in ways that promote and maintain good health [20]. Conversely, low literacy can create misunderstanding of the environment and impact health outcomes [21].

Health literacy requires acquiring information, processing it, and then understanding the data to develop the reasoning required to make the decision. Many studies have shown that literacy level is related to cost and health characteristics like knowledge, health status, and health facilities. However, little work has been done on the health literacy of adolescents and young adults. The literature also shows the association between poor health literacy and adverse health outcomes such as smoking and obesity [22].

The Rapid Estimate of Adult Literacy in Medicine is one of the most accepted tests to assess health literacy. This test is performed by seeing the patient’s potential to pronounce 66 words, including lay terms for parts of the body, illnesses, and common medical words. It is a quick tool that can be administered in less than 2 minutes. As a result, it is one of the most popular tools for assessing health literacy levels. The scoring is divided into 2 groups: 0-44=low health literacy and 45-66=higher health literacy.

#### Well-being Scale

The Ryff scale is a 42-item scale that measures 6 dimensions of well-being and happiness: environmental mastery, self-acceptance, purpose in life, positive relations with others, autonomy, and personal growth [20].

#### Depression, Anxiety, and Stress Scale

A randomized control trial was conducted on young adults to reduce the symptoms of depression with a brief diet intervention. In this study, the DASS-21 tool will be used to assess the symptoms of depression [23].

This scale evaluates depression, anxiety, and stress associated with a negative emotional situation. It is a 42-item self-report questionnaire that has been concise to a scale of 21. It measures on a 0-3 pointing scale, where 0 is for “did not apply to me” and 3 stands for “applicable to me or majority of the time.”

#### Dietary Diversity

In lower-income countries, dietary diversity tools are used to study dietary adequacy. Dietary diversity is a significant problem among poor people as they usually consume starchy staples with or without limited consumption of animal products, fruits, and vegetables [24].

The Food and Nutrition Technical Assistance Diet Diversity score will be computed based on the information obtained regarding consuming several food items within each food group.

#### General Self-Efficacy Scale

It is a 10-item tool validated in Asia [7]. It can also be applied to diverse cultural backgrounds and positively correlated with depression, anxiety, and optimism. To calculate the total score, the total of all items is considered. The total score ranges between 10 and 40, with a higher score indicating more self-efficacy. It is universally accepted and considered an adequate scale to measure self-reported self-efficacy. A relation has been seen in general self-efficacy and social cognitive variables, health behavior, and well-being [25,26].

#### International Physical Activity Questionnaire

The International Physical Activity Questionnaire is considered a validated tool to monitor physical activity among youth and older adults in diverse settings [27].

The participants will complete the questionnaire so that their physical activity can be assessed. The total time spent in physical activity for recreation, occupation, household work, and transportation in the past 7 days will be calculated. The total weekly physical activity (metabolic equivalents of task [MET-hour/week]) will be calculated as the weighted sum of the reported time spent at each intensity using an MET value specific to each category.

#### Data Analysis Plan

The data gathered at baseline will include sociodemographics, KAP (knowledge, attitude, and practice), diet assessment, physical activity, health literacy, social media, and familiarity with technology. Baseline and follow-up data collected will be statistically analyzed.

For continuous and categorical variables, means and SDs and frequencies, respectively, will be calculated. Finally, cross-tabulation of the data from the intervention group against outcome variables will be done to calculate the direction and degree of association in this initial stage. The analysis will be done using SAS v9.3 (SAS Institute), and the results will be reported at 95% CIs and *P*=.049.

### Project Timeline and Milestone

A detailed timeline is presented in Table 1.

**Table 1:** Scheduled timeline of the tasks in the study.

Tasks involved	Timeline (months)							
	1-3	3-5	5-7	7-9	9-15	15-17	17-20	20-22
Focus group discussion and analysis	✓	—^a^	—	—	—	—	—	—
Content creation for the web-based platform	—	✓	—	—	—	—	—	—
Design and development of the web-based platform	—	—	✓	—	—	—	—	—
Heuristic and usability evaluation	—	—	—	✓	—	—	—	—
Data collection	—	—	—	—	✓	—	—	—
Analysis of data	—	—	—	—	—	✓	—	—
Thesis writing and manuscript preparation	—	—	—	—	—	—	✓	—
Dissemination	—	—	—	—	—	—	—	✓

^a^Not applicable.

### Ethics Approval

This study has received ethical approval from the University Research Ethics Committee of Dehradun Institute of Technology University, Dehradun, India (Protocol DITU/UREC/2021/07/9, dated July 21, 2021). The study results will be presented at conferences and webinars to disseminate information to researchers. The results will also be published in national and international scientific journals.

## Results

This study will bring forward the factors that influence the well-being of individuals aged 18-24 years. The 60 in-depth interviews had been conducted by September 30, 2022. Thematic analysis of the qualitative data gathered through the in-depth interviews will be performed. The evidence-based well-being index will be generated for the youth.

## Discussion

### Principal Findings

This research will help understand the factors that influence the well-being of individuals aged 18-24 years. It will also provide in-depth information on the barriers and challenges among youth for well-being. As this is the most critical period of their development, these barriers and challenges will affect their productivity and will indirectly affect their families and community if left unresolved. The findings of this study will help in the design and development of a web-based platform or stand-alone intervention to enhance the well-being of individuals and evaluate the usefulness and effectiveness of the proposed informatics-enabled decision aid platform to improve well-being in the age group of 18-24 years in an Indian setting.

Well-being is the fundamental human goal, which includes feeling good and functioning well [28]. Considering all the variables on which data have been collected, we will develop the well-being index. The World Health Organization-5 Well-Being Index is a simple, short measure of subjective well-being. This scale is used for screening for depression [29] and will also consider other aspects of well-being.

The well-being index generated from this study can be used in other geographic regions for similar populations. If the youth of a nation is healthy, it will lead to the country’s growth.

### Limitations

The first limitation of this study will be the recalling bias as the participants have to fill out the questionnaires on their own. Second, the small sample size will make it difficult to generalize the results on a large number of students.

The study results will be presented at conferences and webinars to disseminate information to researchers. The results will also be published in national and international scientific journals.

### Conclusions

This study will be able to bridge the gap of well-being in college-going students. It is seen that mental health issues are prevalent in low- and middle-income countries due to lack of mental health care. It has a negative impact on the academic performance of university students [30]. As digital health technologies are getting popular and will play a key role in future, it is important that a multidisciplinary approach should be undertaken; only this can bring a change from digital health to digital well-being [31].

The evidence-based digital mental interventions that can be implemented using smartphones or web-based platforms are required for college-going students as they can reduce the existing barriers to getting traditional mental health services that include time and sigma as well [32].

## Abbreviations

DASS: Depression Anxiety Stress Scale
DHI: digital health intervention
MET: metabolic equivalents of task
